# Morphological and nanostructural features of porous silicon prepared by electrochemical etching

**DOI:** 10.1186/1556-276X-7-408

**Published:** 2012-07-20

**Authors:** Hyohan Kim, Namhee Cho

**Affiliations:** 1 Department of Materials Science and Engineering, Inha University, 253 Yonghyundong, Incheon, 402-751, Republic of Korea

**Keywords:** Electrochemical etching, Nanocrystal, Photoluminescence, Porous silicon.

## Abstract

Porous layers were produced on a p-type (100) Si wafer by electrochemical anodic etching. The morphological, nanostructural and optical features of the porous Si were investigated as functions of the etching conditions. As the wafer resistivity was increased from 0.005 to 15 Ω·cm, the etched region exhibited ‘sponge’, ‘mountain’ and ‘column’-type morphologies. Among them, the sponge-type structured sample showed the largest surface area per unit volume. Silicon nanocrystallites, 2.0 to 5.3 nm in size, were confirmed in the porous layers. The photoluminescence peaks varied in the wavelength range of 615 to 722 nm. These changes in the maximum peak position were related to the size distribution of the Si crystallites in the porous silicon. The doping levels of the wafers significantly affect the size distribution of the Si crystallites as well as the light-emitting behavior of the etched Si, which contains nanoscale Si crystallites.

## Background

Silicon has not been considered a proper material for optical device applications owing to its indirect band structure. On the other hand, as luminescence behavior has been observed from chemically etched porous silicon (PS) at room temperature, many studies have examined the origin of the light emitting properties of such indirect band structured semiconductors [1,2]. In addition, PS is considered a strong candidate material for applications in the optoelectronic industries. Moreover, PS can also be used for a variety of sensing systems, such as detecting gasses, pressures and biomedical molecules, as well as anode materials of Li rechargeable batteries owing to its larger surface [3-7]. When Si crystallites embedded in PS become less than approximately 5 nm in size, the luminescence of PS occurs due to the quantum confinement effect [1,2]. Some researchers attribute this to the presence of surface/interface defects [8,9].

### Presentation of the hypothesis

Many fabrication methods have been assessed to produce PS [10]. In particular, electrochemical anodic etching has been the main method for producing PS because electrical variables can be added to the process conditions to obtain better control of the physical and chemical features of the PS. This method has benefits of being simple and economical to produce PS compared to vacuum system-based thin film fabrication facilities, such as e-beam lithography [10].

Some researchers reported the structural variations of PS electrochemically etched under a range of experimental conditions [11-16]. Zhang reported the variation of the morphologies of PS with the relationship between the Si wafers and electrolytes [13]. Depending on the electrical resistivity of Si wafers compared to that of the electrolyte, the formation of pores, such as macro- or micro-pores, can be initiated at a critical current density [17]. The critical current density is changed when the composition and physical characteristics of the electrolytes are varied.

The variation in the morphological features has been investigated with the formation of space charge regions (SCR) in PS [13,16]. The SCR width is varied with experimental conditions such as doping levels, applied biases and current densities. The charge carrier transfer during etching reactions occurs differently depending on the doping levels. In addition to the morphological features, the nanostructural variation with the electrical conditions needs to be examined to understand the optical and electrical properties.

The electrolytes consist of deionized water (DIW), HF and solvents such as ethanol and glycerin [17-20]. The solvent must be completely mixed in the deionized water. Methanol (CH_4_O) ethanol (C_2_H_6_O) and isopropyl alcohol (C_3_H_8_O, IPA) have been frequently used for solvent materials. Especially, IPA has the highest viscosity (2.4 cP) at room temperature and the lowest surface tension (20.8 dyn/cm) among them [21]. So, IPA is considered a proper solvent material to help form a homogeneous-etched surface.

This study investigated the variation of morphological features under a range of etching conditions, such as wafer resistivity and electrolytes. The nanostructural and optical characteristics were examined along with the morphological properties and related to the etching conditions such as doping levels and electrolyte solvents.

### Testing the hypothesis

#### Experimental

Porous silicon samples were prepared by electrochemical etching p-type (100) silicon wafers with a resistivity of 0.005, 1, 4 and 15 Ω·cm. These etched samples are referred to in this manuscript as samples 1, 2, 3 and 4, respectively. The electrolyte consisted of HF, IPA and DIW. To examine the wettability of the electrolyte to Si wafers, the IPA volume fractions of 0 %, 5 %, 33 %, 50 % and 66 % were applied to the etchant. Electrochemical etching was carried out at 50 mA for 30 min. The electrolyte in a Teflon beaker was heated in a temperature-controlled hot plate; the electrolyte temperature was fixed to 40 °C. Platinum, as the contact electrodes, was pasted on the back side to improve the uniformity of the etching current. During the etching process, the electrolytes were agitated to obtain a homogeneous etchant condition near the wafer surface. The etched samples were dried by blowing N_2_ gas (99.999 %) after electrochemical anodic etching.

The optical features were analyzed by photoluminescence (PL; SPEX 1403 He-Cd Laser 325 nm, Horiba Ltd., Tokyo, Japan). The morphology of the etched surface and microstructural features of the PS wafer were examined by scanning electron microscopy (SEM; S-4300, Hitachi, Ltd., Tokyo, Japan) and polarizing microscopy (1161, Nikon Co., Tokyo, Japan). Contact angle analysis (PHX-300, SEO, UT, USA) was performed to examine the wettability of the electrolytes to Si wafers. The nanostructural features were analyzed by X-ray diffraction (XRD, POSCO Beamline 8C1 at Pohang Light Source, Kyungbuk, Korea), Raman spectroscopy (T.64000, Jobin Yvon, NJ, USA), and field emission transmission electron microscopy (JEOL, JEM-2100 F).

#### Morphological features

**Figure 1:**
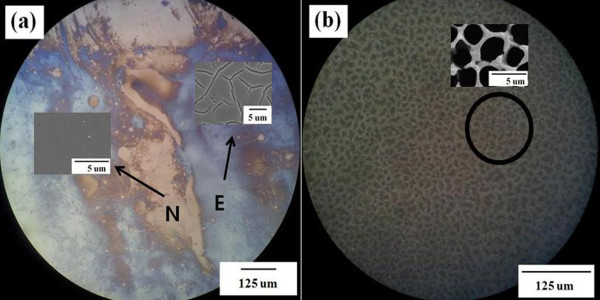
**Polarizing microscopic images of etched wafers.** Micrographs (**a**) and (**b**) were obtained from the samples etched in the etchant with IPA ratios of 0 % and 50 %, respectively. In micrograph (a), region E is a well-etched region, whereas etching reaction rarely occurred in region N. An enlarged image of the marked region is illustrated in the inset.

Figure1 shows optical microscopy images of the etched wafers. The etching reaction occurred heterogeneously when the electrolyte did not contain a solvent (IPA). In Figure1a, a non-etched region was observed on the surface of PS, indicating that some region was not etched or less etched compared to the other regions. In Figure1a, region N appears to be in poor contact with the electrolytes, and as a result, etching did not occur in this region. An approximately 100-μm-wide etched region was neighbored by an approximately 100-μm-wide unetched region. In contrast, in Figure1b, the etching reaction occurred on the Si surface homogeneously, and most of the etched surface consisted of microscale holes; the inset provides details of the column-type surface morphology.

**Figure 2:**
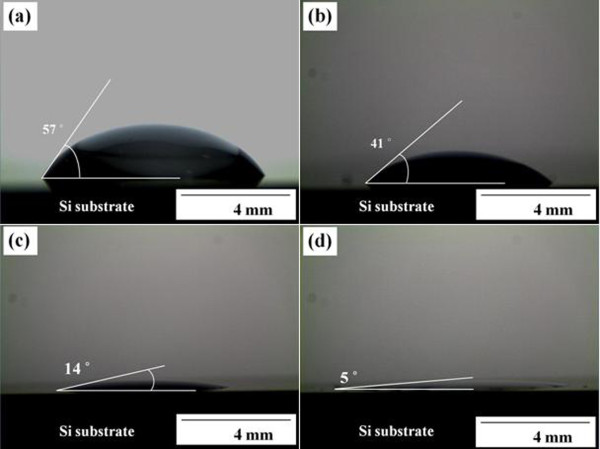
**Contact angle measurement results.** The images in (**a**), (**b**), (**c**) and (**d**) were obtained when a drop of etchant with surfactant (IPA) ratios of 0 %, 5 %, 33 % and 66 % were placed on the surface of the Si wafers, respectively.

The wettability of etchants was examined by measuring the contact angle between the etchant and Si wafers. When the etchant with the highest solvent ratio (IPA, 66 %) was dropped on the Si surface, the etchant spread out rapidly. In contrast, in the case of electrolytes with an IPA ratio of 0 %, 5 % and 33 %, the etchant did not spread but rather stayed like a drop of water on a lotus leaf. When the IPA ratio of electrolytes was increased from 0 % to 33 %, contact angle was varied from 57° to 14° as shown in Figure2a,b,c. In case of IPA ratio of 66 %, it was difficult to measure the contact angle for electrolytes because the etchant spread rapidly, as shown in Figure2d. The variation in the contact angle between the etchant and Si wafer with the IPA contents suggests that a surfactant like IPA plays an important role in generating a homogeneous morphology during electrochemical anodic etching.

**Figure 3:**
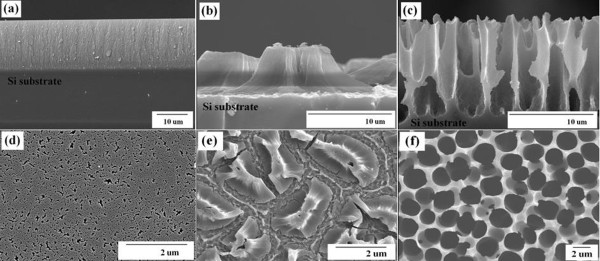
**Cross-section and plane view SEM images.** Micrographs (**a**), (**b**) and (**c**) are cross-section views of the samples with resistivities of 5 × 10^−3^, 4 and 15 Ω·cm, respectively. Micrographs (**d**), (**e**) and (**f**) are plane views of the samples with resistivities of 5 × 10^−3^, 4, and 15 Ω·cm, respectively.

Figure3 shows cross-section and plane view FE-SEM images of the PS. The morphologies of the PS samples 1 to 4 were examined to determine the role of the resistivity of a Si wafer on the etching process. The etching was carried out in electrolytes with the IPA ratio of 50 %. When the resistivity of Si wafer was increased from 0.005 to 15 Ω·cm, the morphology varied from a ‘sponge’ (Figure3d) to a ‘column’ (Figure3f)-type structure.

**Figure 4:**
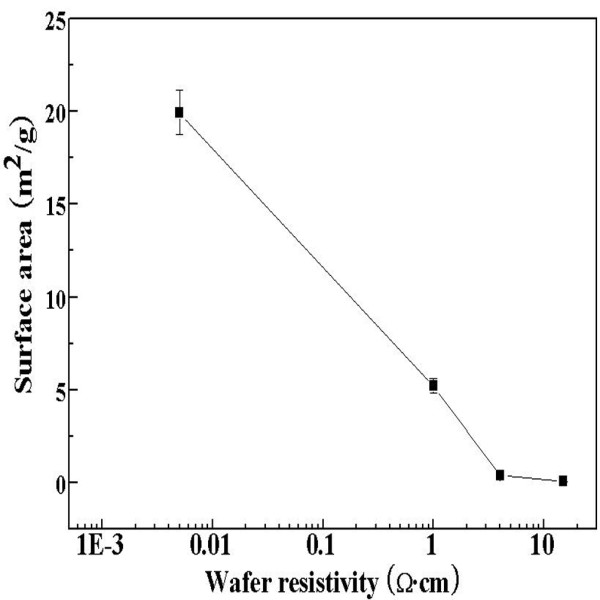
**BET results of the PS.** The change in the surface area per unit volume of the PS as a function of the resistivity of the wafers.

Brunauer Emmett Teller (BET) analysis was carried out to examine the variation in surface area with etched morphologies (Figure4). When the morphology of PS changes from a sponge to column-type structure, the surface area decreased from 19.92 to 0.09 m^2^/g. The sponge-type and column-type structures showed the largest and smallest surface area, respectively.

The silicon wafers with resistivities of 0.005 to 15 Ω·cm are expected to have doping concentrations of 3.62 × 10^19^ to 1.03 × 10^15^ atoms/cm^3^, respectively. The SCR width of the wafers under the etching conditions is critically determined by the distribution of dopants in the wafers. In addition, the charge carrier transfer type is also varied during the etching process with the doping concentrations [22]. As a result, such difference in the doping levels seems to bring up the morphological variation.

#### Nanostructural features

**Figure 5:**
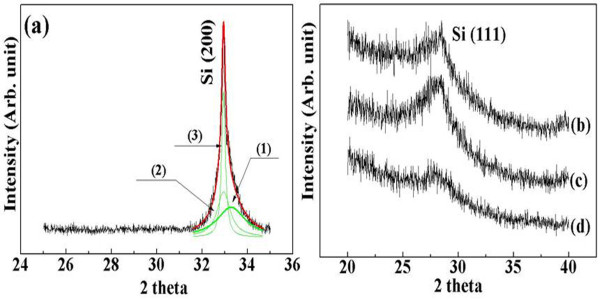
**XRD patterns of porous Si.** XRD patterns (**a**), (**b**), (**c**) and (**d**) were obtained from samples 1, 2, 3 and 4, respectively. In pattern (a), the raw curve is separated into three Lorentzian peaks (1) to (3).

Figure5 shows XRD patterns of the PS samples. The XRD peak at 33° of the sample with a resistivity of 0.005 Ω·cm was assigned to the (200) plane of Si. The raw curve was deconvoluted into three Lorentzian peaks, and the results were used to estimate the nanocrystallite size. The fits of the XRD pattern are in good agreement with the raw data with a reliability of approximately 90 % to 95 %. The mean nanocrystallite size was determined from the FWHM of the XRD peaks using the Scherrer equation [23] as follows:

(1) $$D=\frac{0.9\lambda }{w\cos\text{θ}}\text{,}$$

where *Dλ* and *w* mean the nanocrystallite size, X-ray wavelength and FWHM, respectively. The Si crystallites embedded in the etched layer of sample 1 were 5.3, 16.5 and 96.0 nm, respectively. The relative volume fractions of the crystals with sizes of 5.3, 16.5 and 96.0 nm were 47 %, 40 % and 13 % respectively. Therefore, in sample 1, the crystallite size varied widely; approximately 5-nm-sized crystallites are expected to contribute mainly to the light emitting of the PS at room temperature. Figure5b shows the XRD patterns of samples 2, 3 and 4. The main peaks at 28.4° were assigned to the (111) plane of Si. The Lorentzian function was also applied to examine the nanocrystallite size of these patterns. The nanocrystallite size of these samples ranged from 3.1 to 2.0 nm; the FWHM increased with increasing the resistivity of the Si wafer.

We carried out the grazing incidence X-ray diffraction (GIXD) analysis for samples 2, 3 and 4 [24,25]. In these samples, most nanocrystallites are located on the etched surface, and GIXD analysis is very effective to get good response from the top-etched surface. On the other hand, for sample 1, the thin film X-ray analysis was used to observe the presence of nanocrystallites. This is mainly because about 20-μm-thick etched layer was formed in sample 1, and nanocrystallites are spread all over the etched layer.

**Figure 6:**
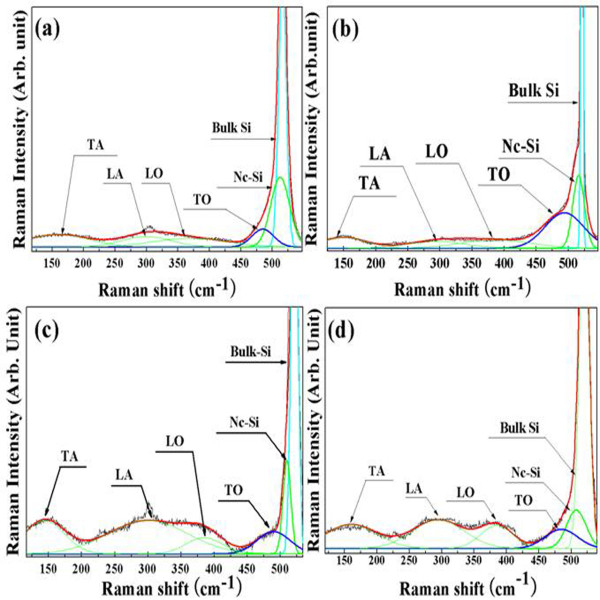
**Raman spectra of porous Si.** Raman spectra (**a**), (**b**), (**c**) and (**d**) were obtained from samples 1, 2, 3 and 4, respectively. The presence of amorphous Si in the etched PS can be confirmed from the transverse optical (TO) mode at 480 cm^−1^ in the spectra; longitudinal optical (LO), longitudinal acoustical (LA) and transverse acoustical (TA) modes are located at 375, 310 and 150 cm^−1^, respectively.

Figure6 shows the Raman spectra of PS samples 1 to 4. The best Gaussian fits of the Raman spectra are shown for each spectrum. The broad peak at 480 cm^−1^ indicates the presence of amorphous silicon, and the shoulder peak at 490 to 520 cm^−1^ was assigned to the presence of nanocrystallite silicon (nc-Si) [26]. The symmetric main peak of bulk Si is centered at 521 cm^−1^[27]. The deconvoluted Gaussian fits provide information on the volume fraction of the nanocrystallites as well as the mean crystallite size. The Equation 2 was used to obtain the mean crystallite size [26,28].

(2) $$\text{D=}2\pi \sqrt{\frac{2.0}{\Delta \text{w}}}\text{,}$$

where Δ*w* is the shift of nc-Si peaks from that of bulk Si (521 cm^−1^). In Figure6, the spectrum fits indicated that bulk Si, nc-Si and amorphous Si co-exist. In Figure6a, the spectrum fit indicated the presence of nc-Si at 517.5 cm^−1^. The nanocrystallite size of sample 1 was estimated to be 4.7 nm. In the case of samples 2, 3 and 4, the fits for the nc-Si spectrum revealed peaks at 516.3, 510.2 and 505.9 nm, respectively. The nanocrystallite size was estimated to be 4.1, 2.7 and 2.3 nm, respectively. The presence of amorphous Si in the etched PS was also confirmed from the TO mode at 480 cm^−1^ in the spectra [27]. The Raman spectra revealed the LO, LA and TA modes at 375, 310 and 150 cm^−1^, respectively.

This observation of the Si nanocrystallite size and distribution by Raman spectroscopy matched the XRD results. The volume fraction of the Si nanocrystallites and amorphous phases in the porous Si was estimated from the Raman spectroscopy results. These values were obtained based on two signals: one (*I*_a_) is the TO mode at 480 cm^−1^, and the other one (*I*_c_) is nc-Si. The relative volume fraction of the crystallites in the PS was calculated using the equation, $Xc=Ic/\left(Ic+\pi Ia\right)$, where *η* is the scattering factor and is regarded as approximately 1.0 for nanocrystallites [29]. The largest volume fraction of the nanocrystallites (approximately 5 nm) was observed for sample 1; the volume fraction of the nanocrystallites was approximately 71.3 %. For samples 2, 3 and 4, the relative volume fractions of the nanocrystallites were 41.7 %, 52.2 % and 50.5 %, respectively. In the Raman results, bulk Si peak is located at 521 cm^−1^. Peaks related to nanocrystallites (490 to 520 cm^−1^) and amorphous (480 cm^−1^) phases were separated from that of substrate bulk Si (521 cm^−1^) with high accuracy (95 % ± 2 %) by deconvolution as shown in Figure6.

**Figure 7:**
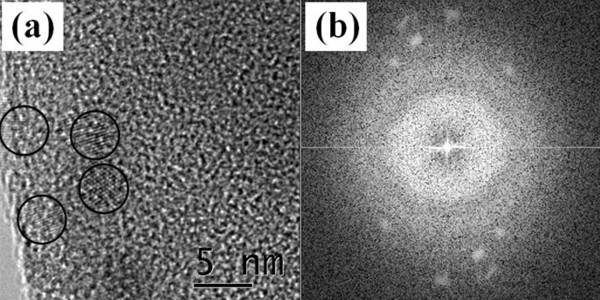
**HRTEM images were obtained from the porous Si.** Micrograph (**a**) was obtained from sample 4. The nanocrystallites are marked with black circles. (**b**) FFT pattern of the image shown in (a).

Figure7 shows a HRTEM image of sample 4. The image reveals an approximately 5-nm sized nanocrystallite embedded in the amorphous matrix. A nanocrystal was marked with black circle in Figure6a. The fast Fourier transform (FFT) diffraction pattern (Figure6b) clearly shows that an etched layer is rather amorphous and contains a small fraction of nanocrystallites.

#### Optical features

**Figure 8:**
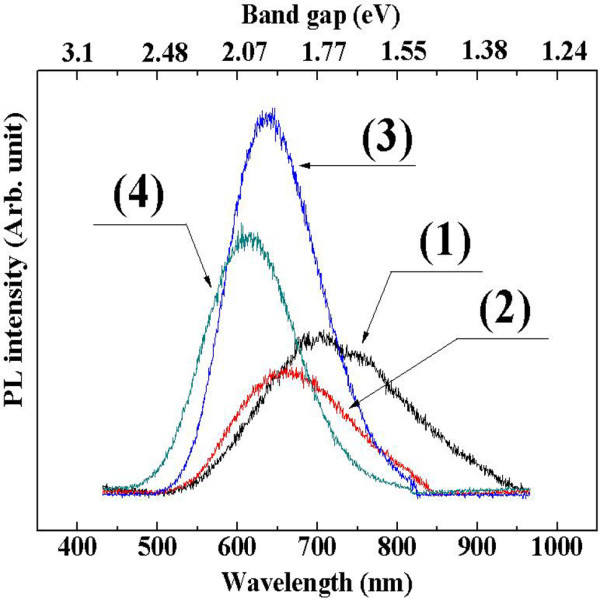
**PL spectra of the porous silicon.** Spectra (1), (2), (3) and (4) were obtained from samples 1, 2, 3 and 4, respectively.

Figure8 shows the PL spectra of the PS samples. In the PL spectra of samples 1, 2, 3 and 4, the maximum peak was observed near 722, 662, 643 and 615 nm, respectively. These wavelengths correspond to the band gap energies of 1.71, 1.87, 1.93 and 2.02 eV. The size of the Si crystallites decreased with increasing wafer resistivity, as shown in the previous nanostructure section, and the optical band gap appeared to increase. This appears to be well matched to the relation of the optical band gap with the Si nanocrystallites, which was reported previously in Blackwood's work [11]. Such correspondence also supports the fact that the observed PL is caused by the presence of nc-Si in the PS.

The maximum intensity of the PL spectra of sample 1 is weaker than those of samples 3 and 4. In addition, the FWHM was asymmetric and wider than the other spectra. This trend was also confirmed by XRD. In the XRD patterns of sample 1 with a sponge-type structure, although the most prominent deconvoluted peak is related to the 5 nm-sized nanocrystallites, there are two other groups of crystallites that can extend the PL to a longer wavelength, as shown in Figure8.

The FWHM of sample 4 is approximately 165 nm, whereas that of sample 1 is about 262 nm. Such a significant difference in size distribution of nanocrystallites between sample 1 and sample 4 might come from the fact that the doping concentration of sample 1 is approximately 10^4^ times higher than that of sample 4. As a result, sample 1 has a larger concentration of doping-related atomic defects, which could be the starting point of anodic chemical etching. On the other hand, in sample 4, the distance between the defects related to doping is much larger than that of sample 1. The nanocrystallites produced in the region between the doping-related defect sites appear to be of approximately 2 nm; approximately 2-nm-sized crystallites must be dominant in the size distribution of sample 4. On the other hand, the distance between such doping-related defects in sample 1 is much smaller than that of sample 4. The dominance of a particular size in the size distribution of nanocrystallites in sample 1 is hardly expected compared to that of sample 4.

From the variation in the size distribution of the nanocrystallites with the doping concentration, it is highly likely that the size of the nanocrystallites is related to the distance from the atomic defects/dopant sites. Under fixed electric and chemical etching conditions, a higher electric current density is to be applied at lattice defects, such as dopant sites, and an etching reaction is expected to start at these sites. The change in electric field strength with the distance from the defects produces a different electric flux, resulting in a particular crystallite size distribution.

The intensity of photoluminescence from sample 1 was lower than those of the samples with ‘mountain’- or column-type surface morphologies. In particular, etching occurred simultaneously along the horizontal and vertical directions on the samples with mountain-type surface morphologies. Nanocrystallites contributing to the photoluminescence were located over the etched surface of sample 3 [30]. In other words, nanocrystallites were spread widely over the etched area and easily contribute to the photoluminescence of sample 2 or 3. In contrast, sample 1 had a wide size distribution, and most were present considerably deep from the surface of the porous layers. As a result, the PL intensity is relatively lower and spreads into the IR region.

Similarly, in sample 4 with a column-like surface morphology, it is evident that vertical etching occurred only after the critical etching depth had been reached, and as a result, most of the nanocrystallites were located at the bottom of the etched column holes. The PS samples with a column-type surface morphology in this experiment showed a 10-μm-etched depth with most nanocrystallites present at the lower inside of the pores. Owing to such a geometrical reason, the intensity of PL light generated at the lower part of the holes, i.e., nanocrystalline regions, might be reduced through absorption before exiting the top surface of the PS samples. Consequently, the PL intensity of sample 3 with a mountain-type structure was higher than that of the samples with sponge and column-type structures.

### Implication of the hypothesis

The change in the morphological, nanostructural and optical characteristics of PS prepared by the electrochemical anodic etching of p-type (100) Si wafers were investigated as a function of the etching conditions. The wettability of electrolytes was increased with the addition of IPA in the etchant. Therefore, the etchant appears to be in good contact with the wafer surface. As a result, homogeneously etched morphologies could be achieved over the entire surface of PS.

When the Si wafer resistivity was varied, etched surfaces exhibited different types of morphologies, including sponge, mountain and column-type morphologies. The sponge-type structured sample exhibited the largest surface area per unit volume. Silicon nanocrystallites with sizes ranging from 5.3 to 2.0 nm were confirmed in the porous layers.

The PL peaks varied in the wavelength range of 615 to 722 nm. The changes in the maximum peak position as well as the FWHM of the spectra were related to the nanostructural features of PS. In particular, the size distribution of Si nanocrystallites and the PL intensity were discussed in terms of the doping levels of the Si wafers. The doping concentration of the wafers significantly affects the size distribution of Si crystallites as well as the light-emitting behavior of the etched Si, which contains nanoscale Si crystallites.

## Competing interests

The authors declare that they have no competing interests.

## Authors’ contributions

NC conceived of the study and participated in the design of the experimental procedures. HK carried out the etching experiment and drafted the manuscript. All authors read and approved the final manuscript.

## Authors’ information

HK is a PhD candidate under Professor NC in the Department of Materials Engineering, Inha University, Republic of Korea.
